# Practicalities of a reduced volume formulation of a C1-INH concentrate for the treatment of hereditary angioedema: real-life experience

**DOI:** 10.1186/s13223-018-0267-4

**Published:** 2018-10-25

**Authors:** John Dempster

**Affiliations:** Barts Health, Grahame Hayton Unit, Ambrose King Centre, The Royal London Hospital, Whitechapel, London, E1 1BB UK

**Keywords:** Hereditary angioedema, HAE, C1 inhibitor, C1-INH, Reduced volume, Quality of Life, Practicalities

## Abstract

**Background:**

Hereditary angioedema (HAE) due to C1 esterase inhibitor (C1-INH) deficiency is characterized by recurrent swelling attacks that can be life-threatening if left untreated. Prompt treatment is vital during acute attacks; plasma-derived C1-INH (Berinert^®^) is one treatment currently licensed for the intravenous treatment of acute HAE attacks in adults, adolescents and children. A new, volume-reduced formulation, of C1-INH is currently available which aims to reduce the time to treatment, and provide greater convenience to patients and healthcare professionals. Here we compare the clinical experience of the reduced volume 1500 IU vial with multiple 500 IU vials.

**Methods:**

HAE patients treated with C1-INH at the Royal London Hospital were selected to take part in this assessment. Included patients were aged 10–65 with moderate to severe HAE requiring high doses of C1-INH. The practicalities of the reduced 1500 IU vial compared with multiple 500 IU vials were assessed, including preparation and administration time, training to self-administer time and several quality of life aspects.

**Results:**

Twenty-three patients participated in this study. Twenty-one patients were previously treated with C1-INH (Berinert^®^) 500 IU for 1–14 years prior to switching to the 1500 IU vial format, two patients were naïve to C1-INH (Berinert^®^). Preparation and administration of C1-INH (Berinert^®^) 1500 IU was faster than an equivalent dose with multiple 500 IU vials (11 and 17 min, respectively) and also required less time to train to self-administer (45 and 55 min, respectively). Overall, patients rated the 1500 IU vial format higher in all assessed aspects than the 500 IU format, including preparation, administration, training, travel and storage. Nonetheless, reconstitution of the 1500 IU vial was noted more difficult, requiring gentle mixing to fully dissolve prior to intravenous injection. Patients remained stable on C1-INH (Berinert^®^) 1500 IU; two patients switched back to multiple 500 IU vials due to headaches and preference for a larger volume.

**Conclusions:**

The volume-reduced C1-INH concentrate (Berinert^®^) 1500 IU is a practical and convenient alternative to multiple 500 IU vials for the treatment of HAE, which provides patients with more control and independence over their disease owing to a simpler to administer treatment.

## Background

Hereditary angioedema (HAE) is a rare autosomal genetic disorder caused by C1 esterase inhibitor (C1-INH) deficiency (type I) or dysfunction (type II) [1]. C1-INH is a serine protease inhibitor that regulates activation of several inflammation pathways. Specifically, C1-INH deficiency/dysfunction results in an excess production of bradykinin, an inflammatory mediator. As a consequence, patients suffer recurrent severe swelling (angioedema) of various body sites, such as extremities, face, tongue, abdomen and throat, which is characteristic of HAE [1]. While abdominal attacks can be excruciating, laryngeal swelling can result in hypoxic brain injury or even death by asphyxiation [1–3]. Therefore, prompt and straightforward treatment is vital during acute attacks. Furthermore, prophylactic treatment to prevent attacks, and to reduce their long-term frequency and severity, is important for those affected by frequent HAE attacks. Several treatments for acute HAE attacks are available, including plasma-derived C1-INH, recombinant C1-INH, bradykinin receptor antagonist (icatibant) and plasma kallikrein inhibitor (ecallantide) (see Table 1). C1-INH is a highly effective and safe treatment for HAE, which can be used for both acute treatment and prophylaxis [4, 5]. The safety and efficacy of prophylactic C1-INH was first demonstrated in a crossover study; prophylactic treatment with 25 U/kg C1-INH every 3 days for 17 days decreased HAE symptoms scores by > 60% compared with placebo [6]. Further randomized, controlled clinical trials have demonstrated a 50% reduction in the frequency of HAE attacks following twice weekly dosing of C1-INH (1000 IU) [7].

**Table 1:** Summary of HAE treatments

Drug	Administration	Indications	Adverse events [1, 22]
Plasma-derived C1-INH^a^	Intravenous, subcutaneous	Self-administration, acute and prophylaxis^b^	Rare: anaphylaxis or thrombosis^c^ Theoretical: transmission of infectious agent^c^ Uncommon: injection site reaction, hypersensitivity, nasopharyngitis, dizziness^d^
Recombinant human C1-INH	Intravenous	Acute	Uncommon: anaphylaxis
Icatibant	Subcutaneous	Self-administration, acute	Common: local swelling, pain, pruritus at injection site
Ecallantide	Subcutaneous	Acute (only US)	Common: prolonged partial thromboplastin time Uncommon: development of antidrug antibodies, anaphylaxis
Tranexamic acid	Oral, intravenous	Prophylaxis	Common: nausea, vertigo, diarrhea, postural hypotension, fatigue, muscle cramps with increased muscle enzymes Uncommon: thrombosis
Androgens^e^	Oral	Prophylaxis	Common: weight gain, virilization, acne, altered libido, muscle pains and cramps, headaches, depression, fatigue, nausea, constipation, menstrual abnormalities, increase in liver enzymes, hypertension, and alterations in lipid profile Uncommon: decreased growth rate in children, masculinization of the female fetus, cholestatic jaundice, peliosis hepatis, and hepatocellular adenoma

^a^Berinert^®^ (IV), Cinryze^®^ (IV), HAEGARDA^®^ (SC)

^b^Berinert^®^ approved for: self-administration, acute treatment in adults and pediatrics, short-term prophylaxis in adults and pediatrics (only EU); Cinryze^®^ approved for: self-administration, prophylaxis in adults and adolescents, acute treatment in adults and pediatrics (only EU); HAEGARDA^®^ approved (only US) for: self-administration, prophylaxis in adults and adolescents

^c^Intravenous administration

^d^Subcutaneous administration

^e^Danazol, stanozolol and oxandrolone

C1-INH (Berinert^®^) is indicated for the intravenous treatment of acute attacks in adults, adolescents and children. Until recently, C1-INH (Berinert^®^) was only available in 500 IU vial sizes with a recommended dose for the treatment of acute attacks of 20 IU/kg body weight [8]. Treatment of an HAE attack in patients weighing over 25 kg would require multiple 500 IU vials; on average the majority of adults require three 500 IU vials. This results in an extended preparation and infusion time and may delay the time to treatment in life-threatening emergency situations. Furthermore, the World Allergy Organization recommends that patients carry on-demand treatment for two attacks at all times [4]. As a result it may be necessary for patients to carry multiple vials; this may increase the burden of disease and lead to reductions in quality of life. A new formulation of C1-INH (Berinert^®^) which has been licensed in the EU since 2015 aims to reduce the time to treatment, and provide greater convenience to patients and healthcare professionals. The 1500 IU vial is 10-times more concentrated (500 IU/ml instead of 50 IU/ml) and has a lower reconstruction volume (3 ml vs. 10 ml) than the 500 IU vial [8], hence the volume of a standard 1500 IU dose in the majority of adults is reduced from 30 ml to 3 ml. The aim of this report is to detail the clinical experience of the C1-INH (Berinert^®^) 1500 IU vial compared with the 500 IU vial.

## Methods

### Patient selection

Between November 2015 and July 2017 patients treated with C1-INH (Berinert^®^) at the Royal London Hospital were selected to take part in this assessment. Patients with moderate to severe HAE requiring high doses of C1-INH were considered for this study because of the benefits of a reduced volume in these patients. All patients were asked to try the new formulation; none declined. This included four patients using C1-INH (Berinert^®^) subcutaneously off-label. Two pediatric patients and two adult patients were switched to subcutaneous (SC) administration on recommendation from their physician because of difficulties with venous access and poor control of attacks on intravenous C1 inhibitor prophylaxis respectively. Due to the severity of HAE these patients had suffered numerous breakthrough attacks. Physicians considered C1-INH SC to be a more appropriate treatment option in these patients as they felt this would provide better control of the patients’ symptoms, particularly due to the smoother PK-PD profile of SC infusions.

### Data collection

Patient data including age, gender, HAE type and severity, age of diagnosis, and administration details on previous and current treatment were collected. Training procedure (i.e., number of sessions) and time taken to master self-administration was recorded for both 1500 IU and multiple 500 IU vials. Training time did not include training for cannulation, only training time for vial reconstitution. To assess the practicalities of administering C1-INH (Berinert^®^), preparation and administration time for both vial formats (1500 IU vs. multiple 500 IU) was estimated and reported by the patient on several occasions. To assess quality of life patients were asked to complete a self-assessment form and rate in a 5-point Likert scale several aspects of C1-INH (Berinert^®^) 500 and 1500 IU. Key challenges raised by patients were also noted.

### Data analysis

In order to assess differences between multiple 500 IU vials and the 1500 IU vial in administration time, training time and each of the rated aspects pairwise comparisons were performed and analyzed with SPSS15.0 statistical package.

## Results

### Patient characteristics

**Fig. 1:**
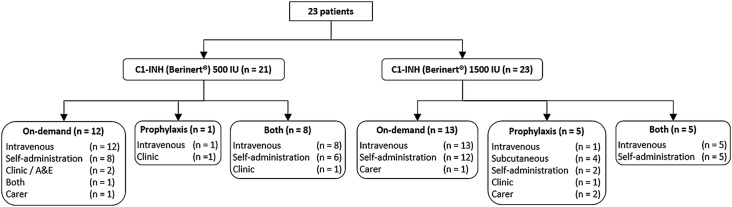
Patient disposition

Twenty-three HAE patients aged 10–65 (37.8 ± 14.7) were assessed. The majority (95.6%) of the patients had type I HAE, the remainder had type II HAE. Twenty-one patients had previously received treatment with C1-INH (Berinert^®^) 500 IU for a mean duration of 6.4 ± 3.9 years (range = 1–14) prior to switching to C1-INH (Berinert^®^) 1500 IU. In patients who switched from multiple 500 IU vials to 1500 IU the total dose was not changed. Twenty out of twenty-one patients decreased the number of vials from three C1-INH (Berinert^®^) 500 IU vials to one C1-INH (Berinert^®^) 1500 IU vial; one patient switched from four C1-INH (Berinert^®^) 500 IU vials to SC administration (3000 IU/dose; two vials). Two patients were naïve to C1-INH (Berinert^®^) treatment and only received the 1500 IU vial format (see Fig. 1 and Table 2 for detailed information). Although all patients remained stable on C1-INH (Berinert^®^) 1500 IU, two patients switched back to C1-INH (Berinert^®^) 500 IU due to headaches and preference for a larger volume.

**Table 2:** Demographics and patients’ information

	N = 23
Age, years, mean (SD)	37.8 (14.7)
Female, n (%)	17 (73.9)
HAE	
Type I, n (%)	22 (95.6)
Type II, n (%)	1 (4.3)
HAE severity^a^	
Moderate, n (%)	12 (52.2)
Severe, n (%)	11 (47.8)
Other HAE treatment before switching, n (%)	23 (100)
Icatibant (alternative to C1-INH for acute attacks)	12 (52.2)
Danazol (prophylaxis)	17 (73.9)
Stanozolol (prophylaxis)	2 (8.7)
Oxandrolone (prophylaxis)	1 (4.3)
Tranexamic acid (prophylaxis)	14 (60.9)
Previous treatment with C1-INH (Berinert^®^) 500 IU, n (%)	21 (91.3)
Adverse effects	0 (0)
Preparation/infusion time, minutes, mean (SD) (n = 18)	16.9 (2.5)
Training time^b^, minutes, mean (SD) (n = 8)	55.6 (6.2)
Years before switching to 1500 IU, mean (SD)	6.4 (3.9)
Current treatment with C1-INH (Berinert^®^) 1500 IU, n (%)	23 (100)
Adverse effects	1 (4.3)
Preparation/infusion time, minutes, mean (SD) (n = 18)	11.1 (2.7)
Training time^b^, minutes, mean (SD) (n = 6)	45 (7.7)
Years current treatment with 1500 IU, mean (SD)	1 (0.49)

^a^Severity scoring criteria [23]

^b^Not including training for cannulation; SD, standard deviation

### Preparation and administration

**Fig. 2:**
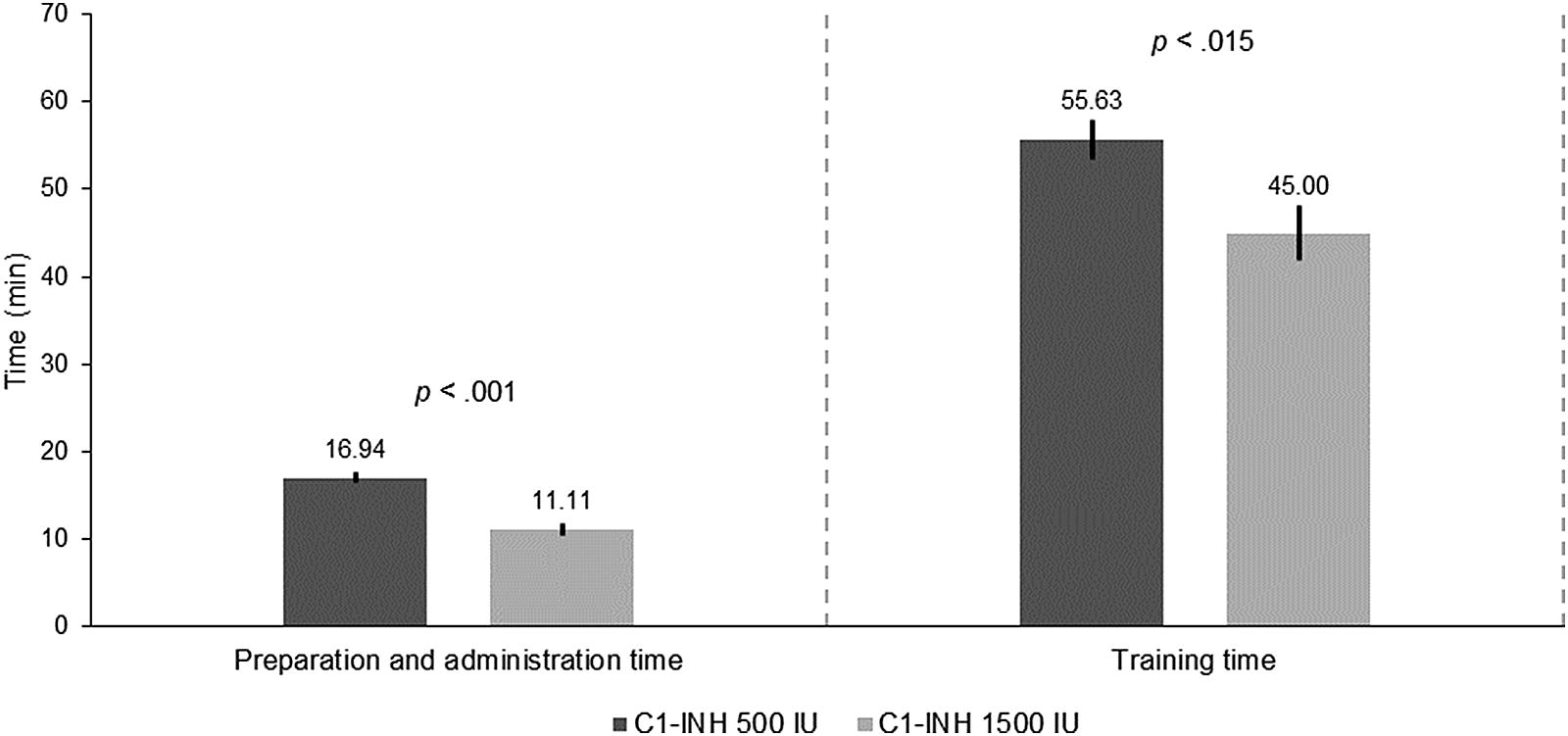
Preparation/administration time and training time required for C1-INH (Berinert^®^) 500 and 1500 IU

Preparation and administration of C1-INH (Berinert^®^) 1500 IU was faster than an equivalent dose with multiple 500 IU vials (mean difference [MD], − 5.8 min; 95% confidence interval [CI], − 7.11 to − 4.55; *t*(17) = − 9.62, *p* < 0.001) (Fig. 2). Patients reported that reconstitution of the 1500 IU vial was more difficult than that of the 500 IU vial; the former took longer to dissolve and required gentle mixing to fully dissolve C1-INH prior to administration. Despite this, reduced preparation intricacy, i.e., preparation and administration of one vs. three syringes/vials, no need to pool multiple vials, and lower infusion times demonstrated the superiority of the 1500 IU formulation.

Differences between vial formats in the time to train to self-administer were assessed by comparing a group of patients that had received training with C1-INH (Berinert^®^) 500 IU (n = 8) to a group of patients naïve to self-administration that received for the first time training with C1-INH (Berinert^®^) 1500 IU (n = 6). Patients required less time to learn to self-administer the 1500 IU format than multiple 500 IU (MD, − 10.6; CI − 18.7 to − 2.5; *t*(12) = − 2.85, *p* < 0.015) (Fig. 2). No additional training was required for 14 patients who switched from multiple 500 IU vials to one 1500 IU vial.

### Patient perspective

Sixteen patients who switched from C1-INH (Berinert^®^) 500 IU to C1-INH (Berinert^®^) 1500 IU rated several aspects of each vial format (Table 3). Overall, patients rated C1-INH (Berinert^®^) 1500 IU higher than 500 IU in all aspects including preparation and administration (Table 3). Patients reported that the administration method was faster to master with the 1500 IU compared with the 500 IU vial size. Patients also rated the preparation and infusion time of a single treatment dose faster with one C1-INH (Berinert^®^) 1500 IU compared with multiple C1-INH (Berinert^®^) 500 IU. Administration of C1-INH (Berinert^®^) 1500 IU was also rated as easier. With regards to convenience, C1-INH (Berinert^®^) 1500 IU vial was easier to store at home and travel with compared with an equivalent dose of multiple C1-INH (Berinert^®^) 500 IU.

**Table 3:** Patients’ ratings of different aspects of the 500 and 1500 IU vial format

Switching patients (n = 16)	500 IU	1500 IU	CI/*t*(15)
Thinking about when you were first trained to use C1-INH (Berinert^®^) 500/1500 IU, how would you rate the experience? (1 very difficult—5 very easy)	3.2 (1.2)	4.69 (0.6)	− 2.17 to − 0.83 − 4.74***
How would you rate the time it took you to master the administration of one treatment dose with C1-INH (Berinert^®^) 500/1500 IU vials? (1 very long—5 very fast)	3.8 (1.1)	4.7 (0.5)	− 1.71 to − 0.67 − 4.84***
How would you rate preparation and infusion time of one treatment dose with C1-INH (Berinert^®^) 500/1500 IU vials? (1 very long—5 very fast)	3.1 (0.9)	4.2 (0.8)	− 1.86 to − 0.38 − 3.2**
How would you rate administration of one treatment dose with C1-INH (Berinert^®^) 500/1500 IU vials? (1 very hard—5 very easy)	3.2 (1.3)	4.5 (0.5)	− 1.88 to − 0.62 − 4.23***
How would you rate storage convenience of C1-INH (Berinert^®^) 500/1500 IU vials? (1 very poor—5 excellent)	3.2 (1.2)	4.9 (0.5)	− 2.38 to − 0.99 − 5.18***
How would you rate travel convenience of C1-INH (Berinert^®^) 500/1500 IU vials? (1 very poor—5 excellent)	2.6 (1.0)	4.7 (0.4)	− 2.75 to − 1.63 − 8.36***
Overall, how satisfied were you with C1-INH (Berinert^®^) 500/1500 IU? (1 very poor—5 excellent)	3.8 (1.1)	4.4 (1.1)	− 1.62 to − 0.37 n.s

Naïve patients (n = 2)		1500 IU	^a^CI/*t*
Thinking about when you were first trained to use C1-INH (Berinert^®^) 1500 IU, how would you rate the experience? (1 very difficult—5 very easy)		3.5 (2.1)	− 17.09 to 19.47 n.s
How would you rate the time it took you to master the administration of one treatment dose with C1-INH (Berinert^®^) 1500 IU vials? (1 very long—5 very fast)		3.5 (2.1)	− 17.43 to 19.55 n.s
How would you rate preparation and infusion time of one treatment dose with C1-INH (Berinert^®^) 1500 IU? (1 very long—5 very fast)		4.0 (0.0)	− 1.09 to 1.47 n.s
How would you rate administration of one treatment dose with C1-INH (Berinert^®^) 1500 IU vials? (1 very hard—5 very easy)		4.5 (0.7)	− 0.84 to 0.84 n.s
How would you rate storage convenience of C1-INH (Berinert^®^) 1500 IU vials? (1 very poor—5 excellent)		4.5 (0.7)	− 0.44 to 1.19 n.s
How would you rate travel convenience of C1-INH (Berinert^®^) 1500 IU vials? (1 very poor—5 excellent)		5.0 (0.0)	− 0.94 to 0.44 n.s
Overall, how satisfied are you with C1-INH (Berinert^®^) 1500 IU? (1 very poor—5 excellent)		4.5 (0.7)	− 1.77 to 1.64 n.s

Mean ratings on a 5-point Likert scale. Standard deviation in parenthesis. CI/t(15), confidence interval of the t test with 15 degrees of freedom

*** p < 0.001; ** p < 0.01; * p < 0.05; *n.s* not significant

^a^Switching vs. naïve patients comparison

No significant differences in patient attitudes were observed between patients who switched from multiple C1-INH (Berinert^®^) 500 IU and those who were naïve to C1-INH (Berinert^®^) 1500 IU treatment (Table 3).

### Subcutaneous, off-label treatment with C1-INH

Four patients received prophylactic treatment with C1-INH (Berinert^®^) 1500 IU via SC injection. Two were pediatric patients (10 year-old males), whose parents administered the subcutaneous treatment, one patient had a dosing regimen of 3000 IU three times a week and the other patient of 1500 IU every 5 days. The other two patients, with severe HAE, self-administered their own treatment, one patient had a fixed dose of 3000 IU three times a week and the other of 3000 IU twice a week. The combined mean preparation and administration time for these patients was 23.75 (7.5) min; this was significantly longer compared with patients that received C1-INH (Berinert^®^) 1500 IU IV (10.53 (1.58) min; CI − 25.09 to − 1.36; *t*(3.06) = − 3.51, *p* < 0.038). The difference in mean preparation and infusion time between IV and SC administration may be due to dosing differences between IV and SC (1500 / dose vs. 3000 IU/dose, respectively) and slightly longer time required to inject SC compared with IV.

### Challenges for treatment with C1-INH

Patients identified numerous challenges for use of both the C1-INH (Berinert^®^) 500 IU and 1500 IU formulations. For the 500 IU vial format the main challenges included the need to carry/utilize multiple vials and change syringes three times during preparation. This was especially challenging for patients to perform when feeling unwell. The primary challenge from a patient perspective for C1-INH (Berinert^®^) 1500 IU was that it took longer to dissolve and required more careful mixing. One patient who switched back to use of three 500 IU vials due to experiencing headaches with the 1500 IU vial, also reported that the 1500 IU vial was more difficult and took longer to dissolve than the 500 IU.

## Discussion

The current study examined the clinical experience with the new C1-INH (Berinert^®^) 1500 IU vial presentation compared with multiple 500 IU vials. The 1500 IU vial format was faster to prepare and administer, and training time for self-administration was easier requiring fewer training sessions compared to the 500 IU vial format. Even though patients noted that the 1500 IU vial took longer to dissolve, the new vial format was rated superior in all assessed aspects of the patient self-assessment form compared with multiple 500 IU vials. Only two patients switched back to multiple 500 IU vials; this was due to headaches and patients believing that the 1500 IU vial was not as effective as (3×) 500 IU vials.

HAE attacks are associated with increased morbidity and mortality [9]. Attacks of swelling affecting the limbs can be disabling (e.g., inability to walk or use hands), and swelling of the face may temporarily disfigure patients, often confining them at home and hampering their work/schooling, social and personal life. Furthermore, patients with severe and frequent abdominal attacks may require narcotics to manage the excruciating pain; this, if uncontrolled, can lead to a narcotic addiction [9]. Attacks often disrupt patients’ life, for instance missing social/family events, impeding travel and leisure activities, increasing absenteeism and reducing productivity, hindering career and educational advancement, even in some cases causing loss of employment [10, 11]. Furthermore, the constant fear of an attack, with the possibility of asphyxiation, severely impairs all aspects of these patients’ quality of life (professional, social, personal, physical and mental) [10–13]. Accordingly, a reduction in attack duration and frequency, and the time to onset of relief has been reported when treatment was administered early and quickly [14, 15]. This, together with access to medication and the ability to self-administer have a large positive impact on patients’ quality of life and mental health (i.e. reducing anxiety and depression) [10, 11, 16, 17]. Therefore, effective, easy and fast to administer treatment is needed to allow patients to manage the severity and duration of their HAE attacks.

HAE is a debilitating chronic disorder that inflicts massive strain in patients’ life; efforts to lessen any factors contributing to the burden of the disease should be sought. Efforts to improve patients’ quality of life have included self-administered C1-INH. Implementation and acceptance of self-administration has been widespread amongst physicians, nurses and patients [18–20]. The results from an online survey reported the experience of ten centers (eight in Europe, one in Canada and one the USA) in implementing and training C1-INH self-administration [18]. Overall, both patients and clinicians agreed that switching to self-administration significantly improved quality of life, independence and convenience [18]. Self-administration allows patients to take control of their disease; they feel less restricted and, due to the ability to administer treatment quickly when- and wherever required, have a reduced fear of attacks [17, 21]. The Berinert^®^ 1500 IU vial presentation has been shown here to be easier to learn to self-administer, and as only one vial is needed, it avoids having to change syringes during administration, thus making self-administration even more convenient. Subsequently, attack-related costs derived from travel expenses to the clinic and medication related paraphernalia (i.e., syringes) [10], and environmental costs from multiple non-recyclable packages and materials could be reduced with the 1500 IU vial. In addition some patients have been able to use the 1500 IU vial for SC injection. This may further increase patients’ conveniences by providing an alternative prophylactic self-treatment option for patients who find IV administration difficult, have poor venous access or are afraid of IV needles.

One limitation of the present study is the small sample size. While this may weaken the results from the subgroup analyses, this study succeeded in gathering and reporting data from 23 patients with a very rare genetic disorder. Another limitation of the study is that it presents self-reported data gathered using questionnaires. While self-reported data may be subjective and difficult to independently verify, it represents the real-life experience of patients. Furthermore, the combination of qualitative and quantitative data reported are mutually supportive.

## Conclusions

The new C1-INH (Berinert^®^) 1500 IU vial presentation is a practical and convenient alternative to the 500 IU formulation for the treatment of HAE. It has demonstrated reduced training and time to treatment (preparation and infusion times) compared with multiple C1-INH (Berinert^®^) 500 IU vials. From a patient perspective the new vial presentation offers a faster and easier to administer treatment, which is more convenient to store and travel with. Therefore, it contributes towards the continued efforts to improve treatment and convenience for HAE patients, further facilitating access to treatment and self-administration, which provides patients with more control and independence.

## Abbreviations

HAE: hereditary angioedema
C1-INH: C1 inhibitor
IU: international units
IV: intravenous
SC: subcutaneous
MD: mean difference
CI: confidence interval
